# Supplementary material for: Corporate social responsibility and firm performance nexus: Moderating role of CEO chair duality
Source: PLoS One. 2023 Aug 3;18(8):e0289037. doi: 10.1371/journal.pone.0289037 (PMC10399786; doi:10.1371/journal.pone.0289037)
Supplement: Attachment — Submitted filename: Response to Reviewers.docx [file pone.0289037.s002.docx]

**RESPONSE TO THE VALUABLE COMMENTS OF THE ACADEMIC EDITOR AND REVIEWERS**

**Corporate social responsibility and firm performance nexus: moderating role of CEO chair duality**

**Reference: PONE-D-23-13630**

---------------------------------------------------------------------------------------------------

The authors would like to thank the Editor, for the opportunity to revise the original manuscript. We also thank the anonymous reviewers for providing us with valuable comments and suggestions, which have improved the quality of the paper. We have examined all the general and specific comments provided by the reviewer and accordingly made the necessary changes to meet their suggestions. We have presented below our responses to reviewer’s query.

We hope that these revisions have improved the quality of the present version of the manuscript and will earn the satisfaction of the reviewer.

---------------------------------------------------------------------------------------------------

**RESPONSE TO ACADEMIC EDITOR**

**Comment # 1:**

Please include the following items when submitting your revised manuscript:

- A rebuttal letter that responds to each point raised by the academic editor and reviewer(s). You should upload this letter as a separate file labeled *'Response to Reviewers'*.
- A marked-up copy of your manuscript that highlights changes made to the original version. You should upload this as a separate file labeled *'Revised Manuscript with Track Changes'*.
- An unmarked version of your revised paper without tracked changes. You should upload this as a separate file labeled *'Manuscript'*.

***Response:***

Thank you. Three separate files have been uploaded as per your suggestion. In the case of *'Revised Manuscript with Track Changes',* changes have been highlighted using coloured text.

**Comment # 2:**

Please ensure that your manuscript meets PLOS ONE's style requirements, including those for file naming.

***Response:***

In the revised version, the PLOS ONE's style guide has been followed.

**Comment # :3**

Please ensure that you include a title page within your main document. Could you therefore please include the title page into the beginning of your manuscript file itself, listing all authors and affiliations.

***Response:***

Title page has been included into the beginning the manuscript file as per instructions.

**Comment # 4:**

We note that you have indicated that data from this study are available upon request. PLOS only allows data to be available upon request if there are legal or ethical restrictions on sharing data publicly. In your revised cover letter, please address the following prompts:

a) If there are ethical or legal restrictions on sharing a de-identified data set, please explain them in detail (e.g., data contain potentially sensitive information, data are owned by a third-party organization, etc.) and who has imposed them (e.g., an ethics committee). Please also provide contact information for a data access committee, ethics committee, or other institutional body to which data requests may be sent.

b) If there are no restrictions, please upload the minimal anonymized data set necessary to replicate your study findings as either Supporting Information files or to a stable, public repository and provide us with the relevant URLs, DOIs, or accession numbers.

We will update your Data Availability statement on your behalf to reflect the information you provide.

***Response:***

The dataset used in this study has been uploaded as Supporting Information file.

**Comment # 5:**

PLOS requires an ORCID iD for the corresponding author in Editorial Manager on papers submitted after December 6th, 2016. Please ensure that you have an ORCID iD and that it is validated in Editorial Manager. To do this, go to ‘Update my Information’ (in the upper left-hand corner of the main menu), and click on the Fetch/Validate link next to the ORCID field.

***Response:***

Thank you for providing guidance. The ORCID iD of corresponding author has been linked with the Editorial Manager.

**Comment # 6:**

Please review your reference list to ensure that it is complete and correct.

***Response:***

In the revised version, reference list and in-text citations have been carefully matched and corrected. Moreover, to manage the consistency in reference list and in-text citations, EndNote has been used.

**RESPONSE TO REVIEWER # 1**

**Comment # 1:**

This article is of great interest to the empirical literature published so far, as it deals with a highly topical and relevant subject. The authors have made a great effort, and I congratulate them on their work. The article is solid and responds to the stated objectives. The conclusions contribute to future articles that may be published in this area.

***Response:***

We would like to thank you for this positive comment.

**Comment # 2:**

The literature review section should be updated with references closer to the present day. That is why I suggest these articles to the authors, which I believe could be of great help to you:

1. Gimeno-Arias, F., Santos-Jaén, J. M., Palacios-Manzano, M., & Garza-Sánchez, H. H. (2021). Using PLS-SEM to analyze the effect of CSR on corporate performance: The mediating role of human resources management and customer satisfaction. An empirical study in the Spanish food and beverage manufacturing sector. Mathematics, 9(22), 2973
2. Palacios-Manzano, M., León-Gomez, A., & Santos-Jaén, J. M. (2021). Corporate Social Responsibility as a Vehicle for Ensuring the Survival of Construction SMEs. The Mediating Role of Job Satisfaction and Innovation. IEEE Transactions on Engineering Management.
3. León-Gómez, A., Santos-Jaén, J. M., Ruiz-Palomo, D., & Palacios Manzano, M. (2022). Disentangling the impact of ICT adoption on SMEs performance: the mediating roles of corporate social responsibility and innovation. Oeconomia Copernicana, 13(3), 831–866. doi:10.24136/oc.2022.024
4. Santos-Jaén, J. M., León-Gómez, A., Ruiz-Palomo, D., García-Lopera, F., & Valls Martínez, M. del C. (2022). Exploring Information and Communication Technologies as Driving Forces in Hotel SMEs Performance: Influence of Corporate Social Responsibility. Mathematics, 10(19), 3629. <https://doi.org/10.3390/math10193629>
5. Ortiz-Martínez, E., Marín-Hernández, S., & Santos-Jaén, J.-M. (2023). Sustainability, corporate social responsibility, non-financial reporting and company performance: Relationships and mediating effects in Spanish small and medium sized enterprises. Sustainable Production and Consumption, 35, 349–364. <https://doi.org/10.1016/j.spc.2022.11.015>
6. Becerra-Vicario, R., Ruiz-Palomo, D., León-Gómez, A., & Santos-Jaén, J. M. (2023). The Relationship between Innovation and the Performance of Small and Medium-Sized Businesses in the Industrial Sector: The Mediating Role of CSR. Economies, 11(3), 92. <https://doi.org/10.3390/economies11030092>

***Response:***

Thank you for constructive comment and for suggesting important studies. In the revised version, suggested studies have been included.

Once again, thank you for the detailed and insightful comments. We are, of course, open to any additional suggestions.
